# The Effect of Changes in Cost Sharing on the Consumption of Prescription and Over-the-Counter Medicines in Catalonia

**DOI:** 10.3390/ijerph18052562

**Published:** 2021-03-04

**Authors:** Mario Martínez-Jiménez, Pilar García-Gómez, Jaume Puig-Junoy

**Affiliations:** 1 Division of Health Research, Faculty of Health & Medicine, Lancaster University, Lancaster LA1 4AT, UK; 2 Erasmus School of Economics, Erasmus University Rotterdam, P.O. Box 1738, 3000 DR Rotterdam, The Netherlands; gaciagomez@ese.eur.nl; 3 Tinbergen Institute, Burgemeester Oudlaan 50, 3062 PA Rotterdam, The Netherlands; 4 School of Management (UPF-BSM), Universitat Pompeu Fabra-Barcelona, Balmes 134, 08008 Barcelona, Spain; jaume.puig@upf.edu

**Keywords:** prescription drugs, healthcare financing, public policy, cost-sharing

## Abstract

Many universal health care systems have increased the share of the price of medicines paid by the patient to reduce the cost pressure faced after the Great Recession. This paper assesses the impact of cost-sharing changes on the propensity to consume prescription and over-the-counter medicines in Catalonia, a Spanish autonomous community, affected by three new cost-sharing policies implemented in 2012. We applied a quasi-experimental difference-in-difference method using data from 2010 to 2014. These reforms were heterogeneous across different groups of individuals, so we define three intervention groups: (i) middle-income working population—co-insurance rate changed from 40% to 50%; (ii) low/middle-income pensioners—from free full coverage to 10% co-insurance rate; (iii) unemployed individuals without benefits—from 40% co-insurance rate to free full coverage. Our control group was the low-income working population whose co-insurance rate remained unchanged. We estimated the effects on the overall population as well as on the group with long-term care needs. We evaluated the effect of these changes on the propensity to consume prescription or over-the-counter medicines, and explored the heterogeneity effects across seven therapeutic groups of prescription medicines. Our findings showed that, on average, these changes did not significantly change the propensity to consume prescription or over-the-counter medicines. Nonetheless, we observed that the propensity to consume prescription medicines for mental disorders significantly increased among unemployed without benefits, while the consumption of prescribed mental disorders medicines for low/middle-income pensioners with long-term care needs decreased after becoming no longer free. We conclude that the propensity to consume medicines was not affected by the new cost-sharing policies, except for mental disorders. However, our results do not preclude potential changes in the quantity of medicines individuals consume.

## 1. Introduction

Pharmaceutical spending represents a large share of health care spending. In particular, pharmaceutical expenditure, excluding hospital medicines, accounted for 17% of total health expenditure in the European Union in 2016 [1]. Organisation for Economic Cooperation and Development (OECD) countries extensively use co-payments as cost-containment measures with adjustments for age, chronic diseases and socioeconomic status [2], and co-insurance reforms have been one of the most common measures to control public health spending between 2008 and 2011 [3].

Spain was not an exception to this trend after the 2008 Great Recession. On July 2012, the Spanish Government implemented the Royal Decree Law 16/2012 (RDL 16/2012) in order to increase the sustainability of the Spanish National Health System (SNHS) [4]. In June 2012 the co-payment for outpatient prescription medicines was reformed in depth, with three concurrent types of cost-sharing policies: a new regional temporary co-payment, changes in the national co-insurance scheme and delisting a broad spectrum of medicines [5].

The international empirical evidence suggests that high levels of cost-sharing are associated with a reduction of consumption of prescription medicines and patient medicine expenditure [6,7,8,9,10]. However, a second strand of the literature finds mixed results about the relationship between co-insurance and the number of prescription medicines used for specific types of illness [11,12] which may point out that the evidence on the impact of cost sharing policies could be highly dependent on the type of medicine and the part of the population under study [13,14].

From 1978, the Spanish National Health System (SNHS) has provided quite generous medicine coverage for all Spaniards, with the exception of the civil servants that follow a different coverage and co-insurance scheme. The general co-insurance rate was 40% of the consumer price of medicines for insured patients, only applied to economically working people and their dependents regardless of their socio-economic status, and a lower co-insurance rate of 10% applied to chronic disease medicines. The pensioners and their dependents were exempted from any co-insurance. 

It was not until more than three decades after, in 2012, that the national cost-sharing system was reformed. The Spanish Government implemented the Royal Decree Law 16/2012 in order to increase financial resources for the SNHS system, controlling expenditure, and increasing efficiency. In particular, the reform of the central government co-insurance entailed an end to free medicines for most pensioners, a decrease in the co-insurance for those unemployed without benefits to nil and at the same time an increase in the co-insurance applicable to some of the active population to 50–60%, depending on their level of income. On 1 October 2012, this reform was fully introduced in Catalonia. Table 1 summarizes the changes in cost-sharing for the different groups of individuals. Nevertheless, other concurrent cost-sharing policies were also implemented in Spain: (i) a temporary introduction of a one-euro co-payment per prescription in two Spanish regions (Catalonia and Madrid (this co-payment was introduced in January 2013 in Madrid, and June 2012 in Catalonia with a maximum annual amount per insured person of 61 €/year in Catalonia and 72 €/year in Madrid)) that was suspended in January 2013 by the Constitutional Court; and (ii) the delisting of a group of more than 400 medicines from pharmaceutical public financing in September 2012. The medicines provided by hospitals to inpatient and outpatient patients remained free of charge. 

There is increasing evidence on the effects of the co-insurance in the SNHS on the consumption of medicines. Puig-Junoy et al. [15] analyse the impact on publicly dispensed prescription medicines (sales) of the co-insurance exemption applied to elderly individuals after retirement before the 2012 cost-sharing reforms. They conclude that this uniform exemption increased the dispensation of prescription medicines on average by 17.5%. Until now, five published studies have estimated the impact of the 2012 Spanish cost-sharing reforms on dispensed prescriptions and expenditure: three used aggregate time series and two used individual data. To the best of our knowledge, we are the first ones to estimate the effects on the probability to consume medicines—including those for which a prescription is not required—using individual data. Studies using a before–after time series approach with NHS aggregate data focusing on a short-time period after the reform (around 12 months) find a decline in the number of the SNHS prescriptions and total public expenditures larger than 10%, [16,17]. A third study using aggregate data analyze the effect on the number of daily defined doses (DDDs), including public and private prescription dispensations, until two and three years after the intervention, but only for chronic treatments such as antidiabetics, antithrombotic and agents against obstructive conditions of the respiratory tract [18]. It concluded that the short-term level effect of the intervention was not permanent, as it was accompanied by a change in the growth trend in the post-intervention months, which partly offset the effect on the level. However, the effects of this reform on the population with long-term care needs, who are often elderly patients with chronic diseases undertaking multiple types of medication, remains unexplored.

The two individual studies used administrative databases to analyze the short-term impact of the 2012 reforms on the number of DDDs [19,20]. Hernández-Izquierdo et al. [19] find a reduction in consumption among low-income pensioners during the first year after the intervention in the autonomous community of Canary Islands, partially offset by a previous increase (stockpiling). García-Gómez et al. [20] limit their attention to the impact of the short-term introduction of a capped co-payment of €1 per prescription in Catalonia during half a year. They conclude that a uniform capped low co-payment may give rise to a major reduction in medicine consumption to a much greater extent among those who previously had free prescriptions. 

There is an extensive empirical literature on the impacts of changes to pharmaceutical cost-sharing on the use of prescription medicines. It is worth noting two systematic reviews of the empirical evidence: first, Kiil and Houlberg [6] include 47 studies published between 1990 and 2011 focused on high-income countries; and Sinnott et al. [21] focus on effect of copayments on the adherence to prescribed medicines in publicly insured populations on high-income countries. Kiil and Houlberg [6] conclude that co-payment reduces the use of healthcare services, except for the prevalence of hospitalisations. In addition, they find that the size of the reduction in the use of prescription medicine is heterogeneous among population groups (e.g., the effect is larger for low-income individuals).

Our aim is to assess the effect of the cost-sharing changes implemented in Spain after June 2012 on the propensity to consume prescribed and over-the-counter (OTC) medicines in Catalonia, a Spanish autonomous community with 7.5 million inhabitants. This paper contributes to the previous literature on the impact of these new cost sharing arrangements in Spain in several aspects. First, we use a series of biannual cross-sectional individual health surveys [22,23,24] which allows measuring self-declared consumption (presence or absence of consumption during the last two days) and not only observed dispensation or sales at the pharmacy. Second, we observe consumption propensity for a longer post implementation period (two years), which allows us to verify potential declining effects of the intervention along the time. Third, we examine the impact of the cost sharing schemes not only on the propensity to consume prescription medicines, but also on the propensity to consume OTC medicines. Rational individuals may consider some prescribed and OTC medicines economic substitutes in the drug utilization decision [25]. Therefore, a complete assessment of the effects of any cost-sharing reform should also evaluate whether this substitution takes place. Fourth, we analyse the heterogeneous effects among seven therapeutic groups to shed some light on whether changes in cost sharing may have particular health consequences. Finally, we focus on a subsample with long-term care needs to examine the impact of changes in the coinsurance scheme on this population group. One would expect these individuals to face both an inelastic demand for medicines and higher economic burden of medication—as many of them are elderly with chronic conditions. However, the existing evidence has not investigated to what extent the design of this reform has hindered the consumption of drugs of this frail group. 

## 2. Material and Methods

### 2.1. Study Design and Data

We used data from the Catalan Health Survey (ESCA) [26], a cross-sectional population-based face-to-face survey to adults older than 15 years of age from Catalonia. The ESCA is conducted by the Catalan Health Department in collaboration with the Spanish National Institute of Statistics (INE). It has collected, twice a year since 2010, detailed health information from a representative sample of the Catalan population. Unfortunately, it does not follow the same individuals over time. We used a sample composed by observations from June 2010 to December 2014 (nine cross-sectional waves). This included observations from two years before and after the 2012 reform. We used the exogenous variation of this reform to estimate the effect of a change in the co-insurance rate using a quasi-experimental design. 

As summarized in Table 1 and Table 2, the reform introduced in October 2012 changed the co-insurance rate of different groups differently, depending on their labour market status and income. The anonymized version of the ESCA does not only contain health information, but also basic socioeconomic characteristics that allow us to identify those groups whose co-insurance rate increased, decreased, or remained unchanged. Our control group was the population group whose co-insurance rate remained unchanged (i.e., low-income working population), while the middle-income working population, the low/middle-income pensioners, as well as, the unemployed without benefits are the individuals who comprise our three intervention groups and each of these groups were affected by a different change in the co-insurance rate: (i) middle-income working population faced a ten percentage point increase in their co-insurance rate; (ii) low and middle-income pensioners lost access to free medicines to face a 10% co-insurance rate; (iii) unemployed without benefits benefited from a sharp reduction in the co-insurance rate from 40% to 0%. 

The ESCA contains information that allows us to identify the population in the control and in each of the three intervention groups. In particular, we used information on the occupational status of each individual, the monthly household income as a proxy for the personal individual yearly income, and whether the individual has ever participated in paid employment. This last one is relevant as the cost-sharing exemption only applies for those unemployed whose period of entitlement of benefits has expired, i.e., they have previously worked. We also excluded individuals that could potentially belong to more than one group. First, we dropped students and housemakers since they can belong either to the low-income working population or the unemployed without benefits groups. Second, we restricted our dataset to those aged at least 20, since non-emancipated adults have the same beneficiary status as the insured person of reference. In addition, we performed a sensitivity analysis removing those individuals aged 26 or under since this rule applies to all individuals younger than 26 years old (Royal Decree-Law 16/2012 states that non-emancipated without income and under 26 years old are always in charge of the insured person and, therefore, they belong to the same beneficiary status as the insured person of reference), so they could be incorrectly classified. 

Our sample contained 8850 interviewed individuals; in detail, 1143 respondents in the second half of 2010, 1017 in first half of 2011, 1056 in the second half of 2011, 1,010 in the first half of 2012, 968 in the second half of 2012, 964 in the first half of 2013, 884 in the second half of 2013, 894 in the first half of 2014, 914 in the second half of 2014. 

### 2.2. Outcome Variables

Our outcome variable of interest is the propensity to consume medicines. We then define a binary variable that takes value 1 if the individual reports consuming any type of medicine during the last two days, and 0 otherwise. We define two additional outcome measures based on the prescription status of the medicines: (1) consumption of medicines prescribed by a physician (medicines prescribed by a physician include both those prescribed by NHS physicians and private practice ones. No formal data exist about the extent of private prescriptions; however, informal estimations suggest that private physicians prescribe around 20% of all the prescriptions of the medicines dispensed in pharmacies. Patients paid the full cost of drugs prescribed by private physicians over our entire observation period); and (2) consumption of medicines by own initiative or advised by the pharmacist during the last two days which do not need a prescription, so called OTC medicines.

To explore the heterogeneous effects across therapeutic groups, we consider seven separate categories depending on whether the consumption of the prescribed medicine is for each of the following groups separately: (1) medicines for alimentary tract and metabolism: insulin or diabetes, stomach, laxative and blood-thinning medicines; (2) medicines for cardiovascular system: blood pressure, heart, cholesterol and anti-inflammatories; (3) medicines for genito-urinary system and sex hormones: contraceptive pills, (4) medicines for anti-infective for systematic use: allergy and antibiotics, (5) medicines for nervous system: tranquilizer/sedatives, antidepressants and sleeping pills, (6) medicines for sensory organs: eye and ear issues; and (7) other groups of medicines: dermatological, musculoskeletal system (osteoporosis), and respiratory system (cough and asthma). These variables have been defined using the internationally accepted standard World Health Organisation (WHO) Anatomic Therapeutic Chemical (ATC) classification system [27].

We also restrict our sample to those individuals with long-term care needs. In particular, those who need assistance and/or help to be able to carry out activities of daily living and personal care. Thus we create a variable which takes 1 if the individual has reported either having at least one physical or sensory limitations (including limitation in sight or partial blindness or total; limitation of hearing even with machine or deaf aid; limitation to talk; limitation of communication to write or read; limitation to leave home alone; problems walking; important limitations of movement; limitation to daily activities; dependency on a machine or instrument; difficulties undertaking basic activities of personal care) or having difficulties in undertaking housekeeping tasks; and 0 otherwise.

### 2.3. Statistical Analysis

#### 2.3.1. Primary Analysis

We exploit the exogenous nature of the changes in the co-payment rate across groups of patients induced by the 2012 reform. The primary statistical approach used is a difference-in-difference model, which is commonly accepted for estimating causal relationships in public health settings [28]. The estimated difference-in-difference model is the following:

(1) $$Y_{it}=intercept+\beta_{0}\times {\mathrm{IG}}_{it}+\beta_{1}\times \left({\mathrm{IG}}_{it}\times Pt\right)+\beta_{2}\times D_{t}+{\theta X}_{it}+E_{it}$$

where $Y_{it}$ is the dependent variable (consumption propensity for prescription medicines, OTC medicines, or any of the 7 therapeutic groups of medicines), for the individual *i* during a time period *t*, where *t* is each of the 9 cross-sections. The variable ${\mathrm{IG}}_{it}$ is a dummy variable that identifies the intervention group; the product between ${\mathrm{IG}}_{it}$ and *P_t_* is the interaction between each intervention group and a dummy for being in the post-treated period, i.e., after the second semester of 2012. We control for time effects including the $D_{t}$ variable; $X_{it}$ is a set of socio-economic control variables; $E_{it}$ is the random error term. We compute the models using Stata 15.1 (StataCorp LP., College Station, TX, USA).

We estimate Equation (1) for each of the three intervention groups with and without explanatory socio-economic control variables, and using robust standard errors for the overall population, as well as for the population with long-term care needs. We estimate these regression models for each of the 9 outcome variables separately. Our coefficient of interest ($\beta_{1}$) is the interaction term between ${\mathrm{IG}}_{it}$ and *P_t_* that allows us to capture the impact of the changes in the co-insurance rate for each intervention group. We estimate the parameter of interest in all the regressions using a linear probability model (LPM), as well as, a non-linear estimation method (logistic model) to test the robustness of our estimates to the linearity assumption. Our results are robust to the model (LPM vs. logit). We provide results from LPM regressions in the main text, while the average marginal effects from the logit model are available in the Appendix A (see Table A1, Table A2 and Table A3). 

For comparison, we also perform a before-after analysis and estimate Equation (2) for each of the treatment groups, excluding the control group. This corresponds to a before-after analysis that could be more appropriate if the control group was also affected by the policy.

(2) $$Y_{it}=intercept+\beta_{0}\times Pt+{\theta X}_{it}+\gamma_{1}t+\gamma_{2}t^{2}+\gamma_{3}t^{3}+\lambda_{mt}+E_{it}$$

where $Y_{it}$ is the dependent variable for the individual *i* during a time period *t*, where *t* is each of the 9 cross-sections. Pt is a dummy variable that takes value 1 after the changes in central government coinsurance on 1 October 2012; and $X_{it}$ refers to the set of explanatory variables that include socio-economic characteristics. We control for time effects, including a cubic time trend and a set of dummies ($\lambda_{mt}$; where *m* 1; ...; 12 months), to pick up seasonal effects. Lastly, $E_{it}$ is the random error term. 

The set of explanatory socio-economic control variables is gender, age, social status and educational attainment. We include an interaction between age (dummies for five age intervals: 20–35; 35–50; 50–65; 65–80; and more than 80) and gender (takes value one if the individual is a woman and zero otherwise). Social status is a categorical variable that groups individuals into three categories: Class I: directors, managers and university professionals (base category); Class II: intermediate jobs and self-employed workers; Class III: manual workers. Educational attainment is categorized into three levels: (i) Primary studies/without studies (base category); (ii) Secondary studies; (iii) University studies. We have decided to exclude health information as a control in the main analysis due to potential reverse causality. However, we test the sensitivity of our results in Section 3.4 below. 

#### 2.3.2. Common Trends Assumption

**Figure 1:**
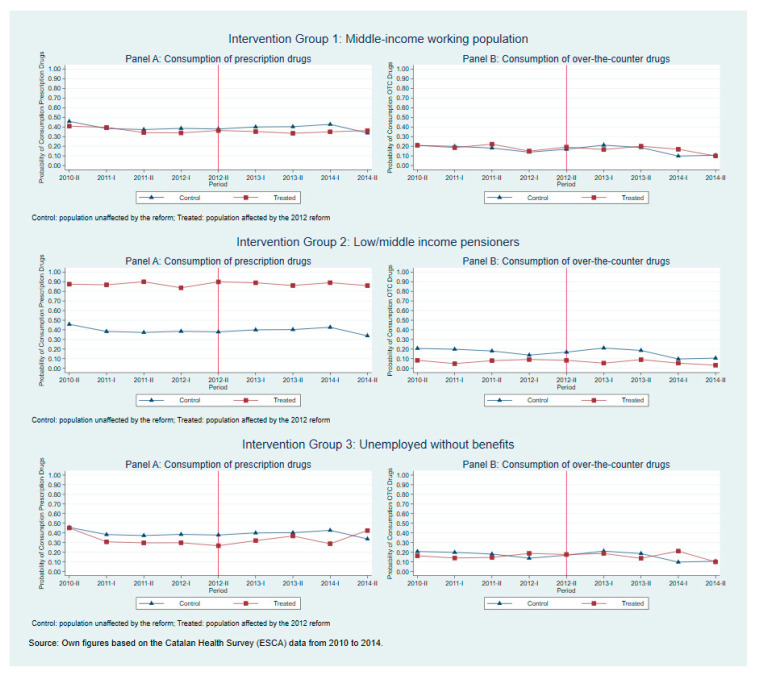
Common trends assumption graphical check.

**Figure A1:**
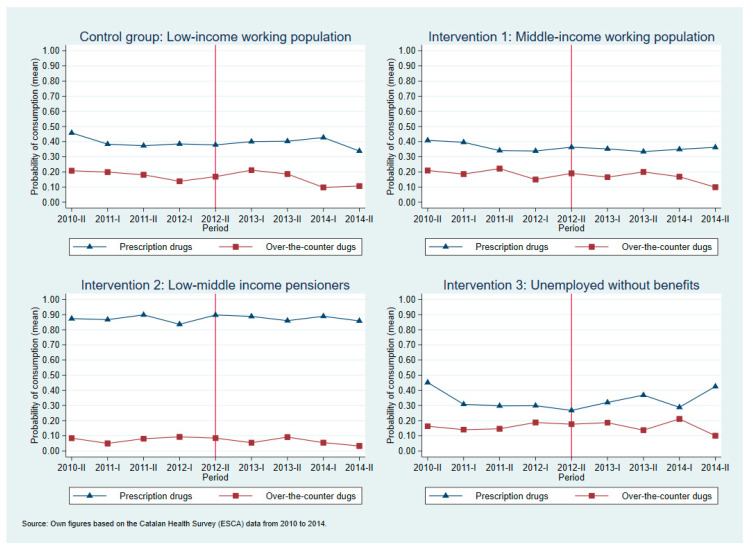
Trends of average propensities to consume medicines during the study period by intervention groups.

The main identifying assumption for $\beta_{1}$ to estimate the effect of the change in the cost-sharing rate on the propensity to consume medicines is the ‘common trends assumption’, which means that trends in the consumption of medicines would have been the same for treatment and control groups in the absence of the change in the co-insurance schemes. While we cannot directly test this assumption, as we do not observe the counterfactual without the reform, we analyse its plausibility for each intervention group both graphically and statistically (the graphics from Figure 1 and Figure A1, the descriptive statistics and the estimations were adjusted with sampling weights that indicated the weight to be attached to each observation. The weighting variable from the ESCA restored the territorial proportionality of the sample.), and obtain evidence in support of using the low-income working population, whose co-insurance rate did not change with the reform as a comparable control group.

Figure 1 plots the trends of the average proportions of the consumption of prescription and OTC medicines during the study period by intervention groups compared to the trends of the control group. These graphs provide support in favour of the common trends assumption since the trends of consumption medicines for each intervention group follow similar patterns than the trends of the low-income working population (control group) for the 4 periods before the 2012 reform. In addition, we estimate Equation (1) using the cross-sectional data before the reform and setting the period January 2011 to June 2011 as a pseudo-intervention period. This formally tests whether the trends were parallel before the reform. Table 3 presents the estimates of interest for each intervention group and outcome variable, being none of them statistically significant. These results provide evidence that the probability to consume medicines followed a parallel trend between each of the intervention groups and the control group before the 2012 reform. 

## 3. Results

Table 4 shows the descriptive statistics of the sample. The average propensity to consume prescription medicines for the low/middle-income pensioners is much higher compared to those of the other two treatment groups both before and after 2012. Also, the average propensity to consume prescribed medicines of middle-income workers decreases from 36.4% to 34.2% after the reform, whereas it increases for pensioners (from 86.1% to 88.4%) and the unemployed (from 29.7% to 33.9%). However, the average OTC consumption of medicines remains very similar for all population groups. 

### 3.1. Impact on Propensity to Consume Prescription and Over-the-Counter (OTC) Medicines

The results in Table 5 show that, although the coefficient of the variable of interest is negative in the case of middle-income workers—who experienced an increase of 10 percentage points in their co-insurance rate–, this effect was not statistically significant. In a similar way, none of the coefficients of interest for low/middle-income pensioners, who did not have free medicines anymore, and unemployed without benefits, who experienced a decrease in their co-insurance rate, is statistically significant. These results suggest that none of the intervention groups affected by the co-insurance reform experienced any significant effect on the overall propensity to consume prescription medicines.

Table 6 reports the estimates for the propensity to consume OTC medicines. The coefficients of the variable of interest follow opposite patterns compared to the case of prescription medicines for almost all the intervention groups, except for low/middle-income pensioners. Nevertheless, none of these coefficients is statistically significant either. Therefore, there is no statistical evidence that new co-insurance rates had a significant effect on the propensity to consume OTC medicines. 

Table A4 shows the estimates for both the propensity to consume prescription and OTC medicines from Equation (2). As before, there is no statistical evidence that new co-insurance rates had a significant effect on the propensity to consume any of both (prescription and OTC) medicines. Therefore, the lack of an effect is not driven by potential spillover effects of the reform on the control group (García-Gómez et al., 2018). 

### 3.2. Impact on Propensity to Consume Prescription Medicines by Therapeutic Group 

Table 7 shows the estimates on the propensity to consume by seven therapeutic groups of medicines. We find that the propensity to consume significantly changes (at 5%) in only two cases. In particular, unemployed without benefits increase their propensity to consume medicines for mental disorders in the last two days by 5.1 percentage points once the co-insurance rate decreased to zero, while the probability to consume other groups of medicines (dermatological, musculoskeletal and respiratory medicines) decreases by 3 percentage points for middle-income workers whose co-insurance rate increased by 10 percentage points. The estimates are similar if we do not include a control group, but perform a before-after analysis (see Table A4). We do not provide results for OTC medicines by therapeutic groups since the number of observations are too small to obtain reliable estimates. 

### 3.3. Impact on the Population with Long-Term Care Needs

We estimate Equation (1) using LPM estimators for all the outcome variables, restricting the sample for those individuals with long-term care needs as described previously. Our findings (see Table 8) suggest that the new coinsurance scheme did not have a statistically significant effect on the probability of consuming prescription medicines of this population group. However, we do find a decrease in the propensity to consume OTC medicines for the middle-income workers, whose co-insurance rate increases 10 percentage points. Finally, we find that low-/middle-income pensioners with long-term care needs decrease their propensity to consume prescribed mental disorders medicines once they have to cost share. This decrease is not compensated for with an increase in OTC. 

### 3.4. Sensitivity Analyses

We check the robustness of our results to six different sensitivity analyses. First, one could be concerned that our conclusions are due to the indirect way in which we define the control group, i.e., based on household income instead of personal income. We then include all working-age individuals in the control group, and estimate the effect for two intervention groups: low/middle-income pensioners and the unemployed without benefits. 

Second, we control for household income in the regression as this can be an important confounding factor. In particular, we control for the following categories: (i) individuals answering “No answer/don’t know” (reference category); (ii) household’s yearly income lower than €18,000; (iii) household’s yearly income between €18,000 and €100,000; (iv) household’s yearly income above €100,000. Third, we test whether our baseline results are partly driven by the euro per prescription co-payment period. In this regard, we estimate the same baseline model as in Equation (1) but without the period when the euro per prescription co-payment was in force (from July 2012 to January 2013 in Catalonia). Fourth, we check whether the effect of the co-insurance reform is dynamic effect, which may lead to smooth the intervention’s effects over time [29]. We estimate a new equation, in which we interacted the intervention groups with a dummy for each of the post-periods. Then, we control for the health status of the respondents. In particular, we control for mental health with a binary variable that takes value 0 for individuals with a normal mental health evaluation and 1 for individuals defined to be at risk of poor mental health using the GHQ-12 index (the mental health variable is measured using a Mental Health Index (GHQ-12) which is derived from the “Golberg General Health Questionnaire”. The GHQ-12 questionnaire includes 12 questions aimed at detecting symptoms of anxiety, depression or insomnia and it moves on a 12-point scale from 0 to 12. A score of more or equal than 6-points is associated with worse mental health. The survey includes a binary version of the GHQ-12 index, which is 0 for individuals with a normal mental health evaluation and 1 for individuals that score high in the GHQ-12 scale and are defined to be at risk of poor mental health. In the analysis, we use this second version of the GHQ-12 –the binary one). We also control for self-assessed health status (SAH) following the classification recommended by the European WHO (1996) (the European WHO (1996) SAH version asked the following question: “Would you say your health is …” and the following response choices: “very good, good, fair, poor, and very poor”), and whether the individual suffers from at least one chronic illness. Last, we estimate the same baseline models but restrict our sample to those individuals older than 26 years, as those aged between 20 and 25 are potentially incorrectly classified.

The results in Table 9 show that none of the intervention coefficients of interest was significant for the propensity to consume prescription and OTC medicines in any of the sensitivity analysis. This is in line with our baseline results. 

## 4. Discussion

This study evaluated how the propensity to consume medicines changed after the cost-sharing reforms adopted in Spain and in Catalonia in 2012. We used individual information on consumption of medicines from several cross-sections of a health survey. At the same time, this was the first study to use a long post-implementation period allowing to estimate mid-term impacts with a difference-in-differences regression design with three intervention groups and individual data, including not only prescription medicines but also OTC medicines, and test the common or parallel trends assumption crucial for the causal interpretation of our results. 

In the base-case analysis, the new cost sharing policy was not effective in changing the overall propensity to consume prescription and OTC medicines for any of the three intervention groups. This implies that the increase of 10 percentage points in the co-insurance rate experienced by the middle-income working population, as well as, the free full coverage experienced by the unemployed without benefits have not had a significant impact on the average propensity to consume outpatient medicines. Moreover, unlike previous articles based on the impact on consumption quantity of medicines [15,16,17,19,20], we also found a non-significant impact after raising the co-insurance rate—from free full coverage to 10%—on the average propensity to consume prescription medicines by pensioners. These previous Spanish studies align with the impact reported previously in the international literature [6]. However, the vast majority of them analysed the impact of cost-sharing changes on the quantity of dispensed medicines but not on self-reported propensity to consume. Our findings indicated that introducing cost-sharing for pensioner individuals who used to receive free medications does not reduce the propensity to consume, except for the group of medicines prescribed for mental disorders. This may indicate that the observed reduction in the quantity of dispensed medicines may be driven by a reduction in the number of dispensed but not consumed medicines (due to stockpiling and over dispensation); and/or by a reduction in the number of consumed medicines but not abandoning the treatment completely, except for the case of mental health treatments. Future research should unravel dispensation reductions between reductions in adherence (to appropriate and non-appropriate treatments), and reductions in over dispensation due to unnecessary stockpiling.

Other studies indicated that the average number of monthly-defined daily dose (DDDs) per individual had been lower among low-income pensioners—those individuals whose co-insurance moved from free full coverage to 10%—after the 2012 reform. However, the results from these studies are not comparable with those of this paper mainly because the nature of the dependent variable. In particular, in this paper, we measured self-declared propensities to consume (a binary variable) rather than the total number of prescriptions or DDDs dispensed or sold, which is the measure most commonly used in this literature. The number of dispensations or sales may be different from effective consumption if stockpiling before the intervention is important. It may also be different from the true measure of consumption changes when the intervention affects the ratio between effective consumption and dispensation or sales.

This paper contributes to the literature including the analysis of the consequences of cost-sharing changes on the consumption propensity for therapeutic groups of medicines. Our results showed that a high decrease in the co-insurance—from 40% to free full coverage—led to a significant increase in the average propensity to consume medicines for mental disorders, including antidepressant and sleeping pills. Along the same lines, we found that an increase of 10 percentage points in the co-insurance rate, from 40% to 50%, led to significant decreases in the average propensity to consume other therapeutic medicines, such as dermatological and respiratory medicines. These results may help indirect conclusions to be drawn about the health consequences of co-insurance changes. These findings are consistent with the previous literature reporting heterogeneous results depending on the therapeutic groups of medicines [5,13,19]. Our findings finally suggested that elderly individuals with long-term care needs decrease their propensity to consume prescribed mental disorders medicines after becoming no longer free, suggesting potential adverse health effects for these individuals and/or a worsening of the quality of life of their informal carers. The extent to which there are medium or long-run effects remains a hypothesis for further research. 

We extended the previous international evidence, which has estimated how cost-sharing changes affects the use of prescription medicines by type of medicines [11,30], focusing on the effects on the propensity to consume prescribed and OTC drugs for a specific population group (older dependent adults). 

One of the limitations of our analysis was that we observed an increase in the average propensity to consume prescription medicines in the period just before to the cost-sharing announcement for those treated groups whose co-insurance increase (see Figure A1), i.e., similar to a stockpiling effect [15,19,31]. Second, the number of observations in our analysis was lower compared to studies that use administrative datasets. However, the use of survey data allowed us to observe not only consumption of prescription medicines but also consumption of OTC medicines. Moreover, it allowed us to measure consumption instead of purchases of medicines. In our opinion, these are two important dimensions that provide complimentary evidence to the existing studies. Also, the binary nature of our outcome measure is informative about the propensity to consume, but not about the quantity to consume (intensity). We cannot dismiss the possibility that despite the fact of not observing significant changes in the overall propensity to consume, the quantity of consumed medicines could have changed after the intervention. Finally, the consumption of medicines prescribed by a physician includes those prescribed by NHS physicians and those prescribed by private practice physicians. However, only those prescriptions prescribed by NHS physicians are liable to co-payment, while the patient supports the full cost of those prescribed by a private practice physician. Therefore, our dependent variable suffered from measurement error. There are no official data about the number of private physicians’ prescriptions, but all evidence suggests that it is around 20% of the prescriptions. 

As happens with other studies using dispensation or sales as the outcome measure, our results did not allow us to infer any implication about patient adherence to treatments [18,32]. Despite the relevance for policy design of other aspects such as the impact on the paediatric population, the substitution effect by therapeutic groups or the different effects according to age, the sample size of our data did not allow us to investigate them any further. This should be addressed in future research.

Notwithstanding these limitations, the results of this paper allow us to argue that the cost-sharing reforms implemented in Spain in 2012 did not significantly affect the overall propensity to consume medicines and only some changes have been observed for two therapeutic groups in selected population groups. These results may seem contrary to the evidence in Hernández-Izquierdo et al. [19], who report a reduction on the average number of monthly DDDs after the reform in another Spanish region (Canary Islands). However, they show that this reduction was mainly driven by the exclusion from pharmaceutical coverage of some medicines (delisting) rather than by the co-insurance increase itself. These results call, then, for caution regarding the conclusions of previous studies that point to a short-negative impact of the cost-sharing reform using dispensation data as the outcome measure. 

## 5. Conclusions

The cost-sharing reforms implemented in Spain and Catalonia in 2012 did not result in a significant change in the propensity to consume overall prescription and OTC medicines, except in the case of mental disorder medicines for the unemployed without benefits who obtained gratuity in access to prescription medicines, and the case of other medicines (such as asthma) for middle-income workers who moved from a 40% to 50% co-insurance rate. Although previous literature indicates that those changes introduced in the Spanish cost-sharing system resulted in a drop-in dispensation, our results point out that this drop was not driven by a decrease in the propensity to consume prescription medicines, except in treating mental health disorders, but rather a drop in the number of doses. These results do not preclude a reduction in adherence but clearly indicates that there is no evidence of the abandon of treatment after the policy intervention. This implication should be considered by policy-makers in order to design more efficient and evidence-based cost-sharing schemes. Policy-makers should be aware that the impact on the propensity to consume medicines is different among some therapeutic groups of medicines as well as population groups when developing their cost-sharing policy strategy. Finally, it is important to keep in mind that changes in the cost-sharing system is only one of several instruments with the potential to reduce the excess of medicine consumption and the consequential expenditure in the health care system. Our results show that, overall, this reform did not significantly change the propensity to consume prescription medicines. Therefore, politicians considering increasing the co-insurance rate as a means to lower health expenditures also need to consider the role of other agents, such as general practitioners, specialist, hospitals or the pharmaceutical industry, given their involvement in the decision making and use of resources related to the national health care system.

## Figures and Tables

**Table 1:** National and Catalan cost-sharing schemes for medicines in Spain. Comparison before and after 2012 national reforms.

Population Group	Catalonia: Euro Per NHS Prescription from 23 June 2012 to 15 January 2013 with an Annual Upper Limit	National Level: Co-Insurance Rates Until July 2012	National Level: Changes in Medicine Co-Insurance Rates after July 2012	National Level: Co-Insurance with an Annual Ceiling after July 2012
Non-contributory and disability pensioners	No	0%	0%	NA
Pensioners with income ≤ €100,000	Yes		10% *	Yes
Pensioners with income > €100,000	Yes		$60\%{\;}^{†}$	Yes
Unemployed without benefits	Yes	40%	0%	NA
Working population with income ≤ €18,000	Yes		40%	No
Working population €18,000 < income ≤ €100,000	Yes		50%	No
Working population with income > €100,000	Yes		60%	No

Note: In the case of low co-insurance medicines, all users with a positive co-insurance rate as of July 2012 pay 10%, with a maximum amount per prescription. Until June 2012, only active workers and their beneficiaries had to pay this 10% rate, also with a maximum amount per prescription. * with a monthly contribution ceiling between €8 when their income is lower than €18,000, and €18 when their income is between €18,000 and €99,999. ^†^ with a monthly contribution ceiling of €60. NA: not applicable as these groups were exempted from co-insurance after July 2012.

**Table 2:** Co-insurance rate changes for control and intervention groups in Catalonia.

Comparasion Groups	Before July 2012	After July 2012	Characteristics
Control group: Low-income working population	40%	40%	Income ≤ €18,000
Intervention group 1: Middle-income working population	40%	50%	€18,000 < Income ≤ €100,000
Intervention group 2: Low/middle -income pensioners	0%	10%	Income ≤ €100,000
Intervention group 3: Unemployed without benefits	40%	0%	Unemployed without benefits

**Table 3:** Test of common trends assumption using differences-in-differences estimates.

Comparasion Groups		Number of Observations
**Propensity to Consume Prescription Medicines**		
Intervention group 1: Middle-income working population	0.042 (0.028) [0.140]	5187
Intervention group 2: Low/middle-income pensioners	−0.013 (0.032) [0.674]	3703
Intervention group 3: Unemployed without benefits	0.040 (0.060) [0.501]	2735
**Propensity to Consume Over-the-Counter (OTC) Medicines**		
Intervention group 1: Middle-income working population	−0.017 (0.021) [0.395]	5187
Intervention group 2: Low/middle-income pensioners	−0.044 (0.023) [0.387]	3703
Intervention group 3: Unemployed without benefits	−0.025 (0.043) [0.554]	2735

Note: The estimated coefficients refer to an interaction term between intervention group and one period before reform’s implementation. Each coefficient has been estimated separately for each intervention group and each outcome variable. All regression models included an intervention group (IG_it_) dummy, period variable (D_t_), and interaction term between being in the intervention group and period after the reform’s implementation *(*$IG_{it}$*$P_{2}$). Robust standard errors in parentheses. *p*-value significance test in brackets.

**Table 4:** Descriptive statistics.

Variables	Pre-Intervention				Post-Intervention			
Proportions (%)	Middle-Income Workers	Control Group	Low/Middle-Income Pensioners	Unemployed without Benefits	Middle-Income Workers	Control Group	Low/Middle-Income Pensioners	Unemployed without Benefits
**Dependent Variables**								
Propensity to consume prescription medicines	36.4 (1160)	*38.8* *(1236)*	86.1 (2813)	29.7 (304)	34.2 (1172)	*39.1* *(1340)*	88.4 (2286)	33.9 (505)
Propensity to consume OTC medicines)	18.4 (586)	*17.7* *(564)*	7.1 (172)	15.7 (160)	18.37 (630)	*16.9* *(279)*	7.46 (193)	16.8 (250)
**Independent Variables**								
**Gender (%)**								
Female	45.7 (1456)	*47.3* *(1507)*	37.1 (899)	41.3 (422)	45.5 (1570)	*43.8* *(1501)*	38.7 (1001)	41.1 (612)
Male	54.3 (1730)	*52.7* *(1679)*	62.9 (1523)	58.7 (600)	54.5 (1868)	*56.2* *(1927)*	61.3 (1585)	58.9 (877)
**Age**								
Age average (years)	41.2 (0.338)	*41.2* *(0.479)*	67.1 (0.361)	36.5 (373)	42.1 (0.262)	*42.4* *(0.357)*	67.2 (0.293)	38.0 (0.566)
20–35	28 (892)	*30.7* *(978)*	-	46.0 (470)	25.1 (860)	*24.9* *(854)*	-	40.5 (603)
35–50	49.8 (1587)	*42.1* *(1341)*	-	35.3 (360)	48.7 (1669)	*44.5* *(1525)*	-	41.1 (612)
50–65	21.2 (675)	*25.9* *(825)*	10.6 (257)	15.8 (161)	25.6 (878)	*29.4* *(1008)*	11.9 (308)	16.1 (240)
65–80	0.5 (16)	*0.9* *(29)*	61.2 (1482)	-	0.4 (14)	*0.4* *(14)*	60.6 (1567)	0.3 (4)
80+	0.09 (3)	*0.01* *(1)*	27.5 (666)	-	0.04 (1)	*-*	27.5 (711)	-
**Education level**								
Primary/without studies	6.8 (217)	*22.8* *(726)*	58.6 (1419)	24.2 (247)	4.6 (158)	*15.1* *(518)*	45.7 (1182)	15.6 (232)
Secondary studies	59.1 (1883)	*64.7* *(2061)*	33.4 (809)	63.0 (644)	58.5 (2005)	*73.4* *(2516)*	46.3 (1197)	71.5 (1065)
University studies	34.1 (1086)	*12.5* *(398)*	7.9 (191)	12.8 (131)	36.9 (1265)	*11.5* *(394)*	7.9 (204)	12.9 (192)
**Social class**								
Class I	25.63 (817)	*6.7* *(213)*	13.9 (337)	9.9 (101)	28.9 (991)	*7.9* *(271)*	18.1 (468)	11.9 (177)
Class II	36.82 (1173)	*34.3* *(1093)*	25.9 (627)	24.4 (249)	34.3 (1176)	*30.5* *(1046)*	20.5 (530)	27.2 (405)
Class III	35.87 (1143)	*57.9* *(1845)*	57.8 (1400)	65.7 (671)	36.1 (1238)	*60.4* *(2071)*	61.4 (1588)	60.9 (1024)
**Self-assessed health (SAH)**								
Very good	31.89 (1016)	*23.48* *(748)*	11.20 (271)	26.68 (273)	32.92 (1128)	*24.06* (825)	8.90 (230)	29.46 (439)
Good	59.77 (1904)	*57.70* *(1838)*	51.78 (1254)	59.01 (603)	59.30 (2033)	*59.79* (2050)	54.52 (1410)	53.55 (797)
Fair	7.98 (254)	*16.44* *(524)*	30.80 (746)	12.75 (130)	7.02 (240)	*14.06* (482)	29.60 (765)	12.58 (187)
Bad	0.35 (11)	*0.21* *(7)*	4.37 (106)	1.54 (16)	0.57 (20)	*1.84* (63)	5.45 (141)	4.25 (63)
Very bad	0.01 (1)	*0.22* *(7)*	1.82 (44)	0.02 (1)	0.19 (7)	*0.23* (8)	1.51 (39)	0.16 (2)
**Mental health**								
Risk of poor mental health	9.35 (297)	*16.61* *(529)*	11.42 (277)	20.32 (208)	8.60 (295)	*12.66* *(434)*	8.56 (221)	15.39 (229)
**Chronic illness**								
Suffer at least one chronic illness	77.62 (2473)	*75.64* *(2410)*	78.57 (1903)	67.5 (690)	75.68 (2594)	*74.45* *(2552)*	77.25 (1998)	70.56 (1051)
Number of observations	3186	*3186*	2422	1022	3428	*3428*	2586	1489

Notes: Standard deviation for the continuous variables, and frequencies (N) for the discrete ones in parentheses. OTC: over-the-counter medicines.

**Table 5:** Impact of the intervention on the propensity to consume prescription medicines.

Propensity to Consume Prescription Medicines						
Title	Middle-Income Workers	Middle-Income Workers	Low/Midd.-Income Pensioners	Low/Mid.-Income Pensioners	Unemployed w/o Benefits	Unemployed w/o Benefits
Co-insurance effects	−0.038 (0.028) [0.182]	−0.044 (0.027) [0.108]	0.013 (0.028) [0.637]	0.015 (0.027) [0.572]	0.032 (0.044) [0.458]	0.016 (0.043) [0.712]
Time Period	Yes	Yes	Yes	Yes	Yes	Yes
Individual Controls	No	Yes	No	Yes	No	Yes
Adjusted R-squared	0.002	0.103	0.248	0.318	0.005	0.110
Observations	5187	5112	3703	3639	2735	2675

Note: This table reports the estimated coefficients $\beta_{1}$ in Equation (1) using a Linear Probability Model. Robust standard errors are in parentheses. *p*-value significance test in brackets. All regressions with individual controls include gender/age groups dummies, educational level dummies and social class dummies.

**Table 6:** Impact of the intervention on the propensity to consume OTC medicines.

Propensity to Consume over-the-Counter Medicines						
Title	Middle-Income Workers	Middle-Income Workers	Low/Midd.-Income Pensioners	Low/Midd.-Income Pensioners	Unemployed w/o Benefits	Unemployed w/o Benefits
Co-insurance effects	0.012 (0.022) [0.602]	0.014 (0.023) [0.550]	0.014 (0.022) [0.512]	0.011 (0.022) [0.605]	−0.014 (0.034) [0.332]	−0.021 (0.035) [0.459]
Time Period	Yes	Yes	Yes	Yes	Yes	Yes
Individual Controls	No	Yes	No	Yes	No	Yes
Adjusted R-squared	0.008	0.024	0.027	0.051	0.007	0.025
Observations	5187	5112	3703	3639	2735	2615

Note: This table reports the estimated coefficients $\beta_{1}$ in Equation (1) using a linear probability model. Robust standard errors are in parentheses. *p*-value significance test in brackets. All regressions with individual controls include gender/age groups dummies, educational level dummies, and social class dummies.

**Table 7:** Impact of the intervention on the propensity to consume prescription medicines according to therapeutic group.

Propensity to Consume Prescription Medicines by Therapeutic Groups						
	Middle-Income Workers	Middle-Income Workers	Low/Middle-Income Pensioners	Low/Middle-Income Pensioners	Unemployed w/o Benefits	Unemployed w/o Benefits
Consumption of alimentary and metabolism medicines						
Co-insurance effects	−0.014	−0.015	0.019	0.017	0.027	0.017
	−0.017	−0.016	−0.03	−0.03	−0.025	−0.025
	[0.395]	[0.353]	[0.527]	[0.585]	[0.288]	[0.506]
Adjusted R-squared	0.004	0.061	0.135	0.174	0.007	0.072
Consumption of cardiovascular system medicines						
Co-insurance effects	−0.015	−0.016	0.04	0.048	0.03	0.029
	−0.024	−0.023	−0.03	−0.029	−0.036	−0.035
	[0.536]	[0.502]	[0.191]	[0.101]	[0.430]	[0.415]
Adjusted R-squared	0.007	0.126	0.267	0.331	0.015	0.139
Consumption of genito-urinary system and sex hormones medicines						
Co-insurance Effects	−0.013	−0.014	−0.002	−0.006	0.003	−0.005
	−0.011	−0.011	−0.009	−0.008	−0.017	−0.016
	[0.303]	[0.219]	[0.963]	[0.484]	[0.782]	[0.746]
Adjusted R-squared	0.002	0.117	0.023	0.143	0.004	0.11
Consumption of anti-infective for systematic use medicines						
Co-insurance Effects	0.003	0.002	0.011	0.012	0.008	0.001
	−0.009	−0.01	−0.016	−0.017	−0.016	−0.016
	[0.767]	[0.866]	[0.517]	[0.456]	[0.625]	[0.939]
Adjusted R-squared	0.002	0.007	0.008	0.012	0.005	0.012
Consumption of nervous system medicines						
Co-insurance Effects	0.018	0.017	0.026	0.029	0.052	0.051
	−0.014	−0.014	−0.023	−0.023	−0.024	−0.024
	[0.198]	[0.223]	[0.254]	[0.194]	[0.032]	[0.033]
Adjusted R-squared	0.002	0.048	0.062	0.119	0.003	0.069
Consumption of sensory organs system medicines						
Co-insurance Effects	0.006	0.006	−0.035	−0.036	−0.003	−0.004
	−0.008	−0.008	−0.017	−0.021	−0.012	−0.013
	[0.518]	[0.504]	[0.095]	[0.093]	[0.762]	[0.746]
Adjusted R-squared	0.003	0.019	0.057	0.084	0.006	0.022
Consumption of other therapeutic groups of prescription medicines						
*Co-insurance* *effects*	−0.028	−0.030	−0.014	−0.014	−0.011	−0.014
	−0.013	−0.013	−0.023	−0.023	−0.018	−0.018
	[0.031]	[0.020]	[0.553]	[0.538]	[0.545]	[0.435]
Adjusted R-squared	0.002	0.02	0.041	0.078	0.002	0.02
Time Period	Yes	Yes	Yes	Yes	Yes	Yes
Individual Controls	No	Yes	No	Yes	No	Yes
Observations	5187	5112	3703	3639	2735	2675

Note: This table reports the estimated coefficients $\beta_{1}$ in Equation (1) using a linear probability model. Robust standard errors are in parentheses. *p*-value significance test in brackets. All regressions with individual controls include gender/age groups dummies, educational level dummies and social class dummies. We do not provide results for OTC medicines by therapeutic groups since the number of observations are too small to obtain reliable estimates.

**Table 8:** Results for the subpopulation ‘*dependent*’ individuals and using a LPM.

	Propensity to Consume Prescription Medicines			Propensity to Consume over-the-Counter Medicines		
	Middle-Income Workers	Low/Midd.-Income Pensioners	Unemployed w/o Benefits	Middle-Income Workers	Low/Midd.-Income Pensioners	Unemployed w/o Benefits
Co-insurance effects	−0.043	−0.010	0.234	−0.078	−0.071	−0.061
	−0.119	−0.052	−0.101	−0.097	−0.049	−0.156
	[0.714]	[0.836]	[0.201]	[0.043]	[0.150]	[0.697]
R-squared	0.231	0.22	0.272	0.105	0.083	0.111
propensity to consume prescription medicines by therapeutic groups						
consumption of alimentary and metabolism medicines						
Co-insurance effects	−0.036	0.063	0.103			
	−0.113	−0.082	−0.201			
	[0.749]	[0.438]	[0.610]			
R-squared	0.18	0.081	0.182			
consumption of cardiovascular system medicines						
Co-insurance effects	−0.079	−0.042	0.045			
	−0.121	−0.067	−0.222			
	[0.516]	[0.530]	[0.839]			
R-squared	0.111	0.184	0.256			
consumption of genito-urinary and sex hormones medicines						
Co-insurance effects	−0.028	−0.020	0.054			
	−0.033	−0.014	−0.04			
	[0.392]	[0.166]	[0.182]			
R-squared	0.13	0.067	0.112			
consumption of anti-infective medicines						
Co-insurance effects	0.099	0.011	0.132			
	−0.061	−0.044	−0.133			
	[0.110]	[0.806]	[0.324]			
R-squared	0.087	0.04	0.127			
consumption of mental disorders medicines						
Co-insurance effects	−0.180	−0.028	0.029			
	−0.118	−0.076	−0.201			
	[0.127]	[0.087]	[0.665]			
R-squared	0.154	0.08	0.227			
consumption of sensory organs system medicines						
Co-insurance effects	0.05	0.083	0.019			
	−0.064	−0.065	−0.107			
	[0.439]	[0.318]	[0.859]			
R-squared	0.079	0.067	0.117			
consumption of other therapeutic groups of prescription medicines						
Co-insurance effects	−0.092	−0.065	0.132			
	−0.089	−0.053	−0.081			
	[0.303]	[0.949]	[0.108]			
Pseudo R-squared	0.085	0.066	0.089			
Observations	247	771	149	247	771	149

Note: This table reports the estimated coefficients $\beta_{1}$ in Equation (1) using a linear probability model. Robust standard errors are in parentheses. *p*-value significance test in brackets. All regressions with individual controls include gender/age groups dummies, educational level dummies and social class dummies.

**Table 9:** Results for the sensitivity analysis.

	Propensity to Consume Prescription Medicines			Propensity to Consume Over-the-Counter Medicines		
	Middle-Income Workers	Low/Midd.-Income Pensioners	Unemployed w/o Benefits	Middle- Income Workers	Low/Midd.-Income Pensioners	Unemployed w/o Benefits
(1) Treatment and control groups used						
Co-insurance effects		0.042	0.021		0.014	0.028
		−0.017	−0.033		−0.014	−0.031
		[0.115]	[0.250]		[0.313]	[0.368]
R-squared		0.249	0.249		0.028	0.028
Observations		11,596	11,596		11,596	11,596
(2) Control for yearly income						
Co-insurance effects	−0.043	0.005	0.021	0.013	0.011	0.03
	−0.027	−0.032	−0.043	−0.023	−0.022	−0.035
	[0.116]	[0.879]	[0.630]	[0.557]	[0.608]	[0.391]
R-squared	0.103	0.314	0.108	0.024	0.051	0.028
Observations	5112	3134	2675	5112	3639	2675
(3) Isolating the impact from the Euro per prescription period						
Co-insurance Effects	−0.030	0.016	0.018	−0.004	0.004	0.007
	−0.032	−0.027	−0.047	−0.026	−0.026	−0.038
	[0.351]	[0.551]	[0.697]	[0.889]	[0.878]	[0.854]
R-squared	0.105	0.318	0.108	0.023	0.05	0.027
Observations	4461	3639	2328	4461	3134	2328
(4) Dynamic effects of the co-insurance intervention						
Co-insurance Effects*Period5	−0.023	0.068	−0.025	0.046	0.035	0.088
	−0.043	−0.037	−0.071	−0.037	−0.038	−0.074
	[0.598]	[0.065]	[0.717)	[0.213]	[0.357]	[0.223]
Co-insurance effects*Period6	−0.046	0.011	−0.044	−0.048	−0.053	−0.045
	−0.04	−0.047	−0.067	−0.04	−0.039	−0.061
	[0.323]	[0.815]	[0.502]	[0.229]	[0.180]	[0.457]
Co-insurance effects*Period7	−0.058	−0.018	0.033	0.004	0.012	−0.064
	−0.041	−0.048	−0.067	−0.041	−0.042	−0.053
	[0.212]	[0.706]	[0.602]	[0.923]	[0.771]	[0.229]
Co-insurance effects*Period8	−0.063	−0.007	−0.055	0.057	0.067	0.109
	−0.043	−0.047	−0.067	−0.036	−0.032	−0.058
	[0.191]	[0.868]	[0.409]	[0.123]	[0.124]	[0.059]
Co-insurance effects*Period9	0.04	0.051	0.151	−0.008	0.031	0.005
	−0.042	−0.047	−0.071	−0.034	−0.033	−0.049
	[0.400]	[0.280]	[0.187]	[0.813]	[0.301]	[0.991]
R-squared	0.103	0.314	0.107	0.027	0.054	0.028
Observations	5833	4212	2675	5833	4212	2675
(5) Health controls						
Co-insurance effects	−0.039	−0.001	0.002	0.002	−0.011	0.014
	−0.029	−0.029	−0.046	−0.024	−0.02	−0.039
	[0.172]	[0.960]	[0.972]	[0.928]	[0.651]	[0.717]
R-squared	0.195	0.388	0.222	0.038	0.065	0.222
Observations	4419	3013	2311	4419	3013	2311
(6) Exclude adults younger than 26 years old						
Co-insurance effects	−0.038	0.019	0.02	0.014	0.01	0.026
	−0.028	−0.027	−0.049	−0.023	−0.022	−0.039
	[0.182]	[0.493]	[0.679]	[0.530]	[0.638]	[0.501]
R-squared	0.098	0.309	0.1	0.024	0.053	0.03
Observations	4800	3508	2410	4800	3508	2410
Time Period	Yes	Yes	Yes	Yes	Yes	Yes
Individual Controls	Yes	Yes	Yes	Yes	Yes	Yes

Note: This table reports the estimated coefficients $\beta_{1}$ in Equation (1) using a linear probability model. Robust standard errors are in parentheses. *p*-value significance test in brackets. All regressions with individual controls include gender/age groups dummies, educational level dummies and social class dummies. In the fourth sensitivity analysis, we included the periods after the reform’s implementation interacted with each intervention group that is defined as follows: IGit*Period5 was the interaction between each intervention group and a dummy for being in the period 5 that included observations from October to December 2012; IGit*Period6 was the interaction between each intervention group and a dummy for being in the period 6 that included observation from January to June 2013; IGit*Period7 was the interaction between each intervention group and a dummy for being in the period 7 that included observation from July to December 2013; IGit*Period8 was the interaction between each intervention group and a dummy for being in the period 8 that included observation from January to June 2014; IGit*Period9 was the interaction between each intervention group and a dummy for being in the period 9 that included observation from July to December 2014.

## Data Availability

Restrictions apply to the availability of these data. Data were obtained from Departament de Salut de la Generalitat de Catalunya under a contract that does not allow researchers to share the database with any other third-party.

## Appendix A

**Table A1:** Results for the propensity to consume prescription medicines using non-linear estimation method (logit).

Propensity to Consume Prescription Medicines						
	Middle-Income Workers	Middle-Income Workers	Low/Midd.-Income Pensioners	Low/Mid.- Income Pensioners	Unemployed w/o Benefits	Unemployed w/o Benefits
Co-insurance effects	−0.038 (0.028) [0.181]	−0.044 (0.027) [0.111]	0.024 (0.035) [0.471]	0.026 (0.032) [0.432]	0.033 (0.045) [0.439]	0.023 (0.043) [0.685]
Time Period	Yes	Yes	Yes	Yes	Yes	Yes
Individual Controls	No	Yes	No	Yes	No	Yes
Pseudo R-squared	0.002	0.080	0.200	0.261	0.004	0.088
Observations	5187	5112	3703	3639	2735	2612

Note: Results are from diff-in-diff estimates from non-linear estimation method (logit). We report average marginal effects (AMEs). Robust standard errors are in parentheses. *p*-value significance test in brackets. All regressions with individual controls included gender/age groups dummies, educational level dummies and social class dummies.

**Table A2:** Results for the propensity to consume OTC medicines using non-linear estimation method (logit).

Propensity to Consume over-the-Counter Medicines						
	Middle-Income Workers	Middle-Income Workers	Low/Middle-Income Pensioners	Low/Middle-Income Pensioners	Unemployed w/o Benefits	Unemployed w/o Benefits
Co-insurance effects	0.013 (0.022) [0.572]	0.014 (0.022) [0.523]	0.004 (0.027) [0.879]	0.002 (0.027) [0.932]	−0.014 (0.027) [0.336]	−0.021 (0.029) [0.468]
Time Period	Yes	Yes	Yes	Yes	Yes	Yes
Individual Controls	No	Yes	No	Yes	No	Yes
Pseudo R-squared	0.009	0.026	0.039	0.070	0.008	0.027
Observations	5187	5112	3703	3639	2735	2661

Note: Results are from diff-in-diff estimates from non-linear estimation method (logit). We report average marginal effects (AMEs). Robust standard errors are in parentheses. *p*-value significance test in brackets. All regressions with individual controls include gender/age groups dummies, educational level dummies and social class dummies.

**Table A3:** Results for the propensity to consume therapeutic groups of prescription medicines using non-linear estimation method (logit).

Propensity to Consume Prescription Medicines by Therapeutic Groups						
	Middle-Income Workers	Middle-Income Workers	Low/Middle-Income Pensioners	Low/Middle-Income Pensioners	Unemployed w/o Benefits	Unemployed w/o Benefits
Consumption of alimentary and metabolism medicines						
Co-insurance effects	−0.014	−0.016	0.028	0.027	0.034	0.022
	−0.016	−0.064	−0.027	−0.027	−0.03	−0.03
	[0.368]	[0.321]	[0.316]	[0.322]	[0.264]	[0.446]
Pseudo R-squared	0.006	0.096	0.128	0.176	0.013	0.106
Consumption of cardiovascular system medicines						
Co-insurance effects	−0.016	−0.015	0.036	0.049	0.038	0.036
	−0.024	−0.023	−0.03	−0.029	−0.043	−0.041
	[0.548]	[0.513]	[0.160]	[0.102]	[0.373]	[0.376]
Pseudo R-squared	0.006	0.116	0.203	0.265	0.015	0.131
Consumption of genito-urinary and sex hormones medicines						
Co-insurance effects	−0.012	−0.032	−0.002	−0.004	0.001	−0.008
	−0.011	−0.025	−0.009	−0.009	−0.02	−0.039
	[0.298]	[0.199]	[0.789]	[0.529]	[0.945]	[0.823]
Pseudo R-squared	0.007	0.118	0.01	0.141	0.022	0.124
Consumption of anti-infective medicines						
Co-insurance effects	0.003	0.002	0.012	0.013	0.008	0.003
	−0.011	−0.011	−0.015	−0.015	−0.016	−0.016
	[0.797]	[0.865]	[0.400]	[0.383]	[0.631]	[0.855]
Pseudo R-squared	0.01	0.024	0.021	0.035	0.02	0.048
Consumption of mental disorders medicines						
Co-insurance effects	0.018	0.017	0.037	0.04	0.047	0.045
	−0.014	−0.014	−0.023	−0.023	−0.032	−0.031
	[0.184]	[0.207]	[0.109]	[0.101]	[0.031]	[0.042]
Pseudo R-squared	0.004	0.079	0.072	0.139	0.004	0.107
Consumption of sensory organs system medicines						
Co-insurance effects	0.007	0.008	−0.006	−0.006	−0.001	−0.002
	−0.008	−0.009	−0.02	−0.02	−0.016	−0.016
	[0.410]	[0.404]	[0.747]	[0.783]	[0.924]	[0.893]
Pseudo R-squared	0.013	0.071	0.106	0.145	0.021	0.078
Consumption of other therapeutic groups of prescription medicines						
Co-insurance effects	−0.028	−0.031	−0.007	−0.009	−0.011	−0.016
	−0.013	−0.013	−0.021	−0.021	−0.021	−0.023
	[0.031]	[0.019]	[0.732]	[0.680]	[0.605]	[0.459]
Pseudo R-squared	0.005	0.043	0.062	0.107	0.006	0.049
Time Period	Yes	Yes	Yes	Yes	Yes	Yes
Individual Controls	No	Yes	No	Yes	No	Yes
Observations	5187	5112	3703	3639	2735	2672

Note: Results are from diff-in-diff model from non-linear estimation method (logit). We report average marginal effects (AMEs). Robust standard errors are in parentheses. *p*-value significance test in brackets. All regressions with individual controls include gender/age groups dummies, educational level dummies and social class dummies. We do not provide results for OTC medicines by therapeutic groups since the number of observations are too small to obtain reliable estimates.

**Table A4:** Before-and-after analysis.

	Propensity to Consume Prescription Medicines			Propensity to Consume over-the-Counter Medicines		
	Middle-Income Workers	Low/Midd.-Income Pensioners	Unemployed w/o Benefits	Middle-Income Workers	Low/Midd.-Income Pensioners	Unemployed w/o Benefits
Co-insurance effects	−0.053	0.07	0.097	0.002	0.005	−0.046
	−0.049	−0.047	−0.101	−0.041	−0.041	−0.087
	[0.908]	[0.135]	[0.337]	[0.974]	[0.896]	[0.600]
R-squared	0.095	0.055	0.117	0.025	0.029	0.031
Propensity to consume prescription medicines by therapeutic groups						
Consumption of alimentary and metabolism medicines						
Co-insurance effects	−0.004	0.046	0.012			
	−0.027	−0.071	−0.063			
	[0.899]	[0.516]	[0.848]			
Pseudo R-squared	0.062	0.047	0.061			
Consumption of cardiovascular system medicines						
Co-insurance effects	−0.037	0.096	0.008			
	−0.04	−0.061	−0.086			
	[0.362]	[0.119]	[0.923]			
Pseudo R-squared	0.111	0.041	0.122			
Consumption of genito-urinary and sex hormones medicines						
Co-insurance effects	−0.002	−0.008	−0.006			
	−0.02	−0.034	−0.054			
	[0.938]	[0.785]	[0.685]			
Pseudo R-squared	0.116	0.052	0.075			
Consumption of anti-infective medicines						
Co-insurance effects	0.008	0.027	0.087			
	−0.021	−0.041	−0.036			
	[0.684]	[0.507]	[0.116]			
Pseudo R-squared	0.01	0.02	0.061			
Consumption of mental disorders medicines						
Co-insurance effects	0.018	0.081	−0.029			
	−0.027	−0.061	−0.063			
	[0.501]	[0.183]	[0.043]			
Pseudo R-squared	0.047	0.08	0.094			
Consumption of sensory organs system medicines						
Co-insurance effects	0.004	0.02	0.074			
	−0.017	−0.051	−0.368			
	[0.111]	[0.685]	[0.045]			
Pseudo R-squared	0.027	0.056	0.083			
Consumption of other therapeutic groups of prescription medicines						
Co-insurance effects	−0.009	0.003	0.007			
	−0.021	−0.053	−0.043			
	[0.011]	[0.949]	[0.872]			
Pseudo R-squared	0.023	0.055	0.023			
Observations	3139	1666	702			

Note: Results are from before-and-after estimates. Robust standard errors are in parentheses. *p*-value significance test in brackets. All regressions include gender/age groups dummies, educational level dummies, social class dummies, and a time trend. We do not provide results for OTC medicines by therapeutic groups since the number of observations are too small to obtain reliable estimates.
